# Influence of Post-Processing on the Degree of Conversion and Mechanical Properties of 3D-Printed Polyurethane Aligners

**DOI:** 10.3390/polym16010017

**Published:** 2023-12-20

**Authors:** Luka Šimunović, Antonija Jurela, Karlo Sudarević, Ivana Bačić, Tatjana Haramina, Senka Meštrović

**Affiliations:** 1 Department of Orthodontics, School of Dental Medicine, University of Zagreb, 10000 Zagreb, Croatia; lsimunovic@sfzg.hr; 2 Dental Clinic Fiziodent, 10000 Zagreb, Croatia; dr.toja@gmail.com (A.J.); ksudarev17@gmail.com (K.S.); 3 Forensic Science Centre “Ivan Vučetić”, Ministry of the Interior, 10000 Zagreb, Croatia; ivana.bacic@mup.hr; 4 Department of Materials, Faculty of Electrical Engineering and Computing, University of Zagreb, 10000 Zagreb, Croatia; tatjana.haramina@fsb.hr

**Keywords:** polymerization, mechanical properties, polyurethane

## Abstract

Background: This study explores how different post-processing methods affect the mechanical properties and degree of conversion of 3d-printed polyurethane aligners made from Tera Harz TC-85 resin. Methods: Using Fourier-transform infrared (FTIR) spectroscopy, the degree of conversion of liquid resin and post-processed materials was analyzed. This investigation focused on the effects of various post-curing environments (nitrogen vs. air) and rinsing protocols (centrifuge, ethanol, isopropanol, and isopropanol + water). The assessed mechanical properties were flexural modulus and hardness. Results: The degree of conversion showed no significant variance across different groups, though the polymerization environment influenced the results, accounting for 24.0% of the variance. The flexural modulus varied considerably, depending on both the rinsing protocol and the polymerization environment. The standard protocol (centrifugation followed by nitrogen polymerization) exhibited the highest flexural modulus of 1881.22 MPa. Hardness testing revealed significant differences, with isopropanol treatments showing increased resistance to wear in comparison to the centrifuge and ethanol rinse treatments. Conclusions: This study conclusively demonstrates the adverse effects of oxygen on the polymerization process, underscoring the critical need for an oxygen-free environment to optimize material properties. Notably, the ethanol rinse followed by nitrogen polymerization protocol emerged as a viable alternative to the conventional centrifuge plus nitrogen method.

## 1. Introduction

The advancement of additive manufacturing technology has significantly impacted the field of dentistry, particularly in the fabrication of dental aligners [1,2]. Shape-memory polyurethane aligners, known for their flexibility and strength, have emerged as a popular choice in orthodontic treatment due to their ability to return to a pre-defined shape when exposed to specific stimuli [3,4]. The conversion rate during the additive manufacturing process, a critical factor in determining the aligner’s mechanical properties, is highly influenced by the post-processing protocols [5,6,7,8]. These protocols, including various rinsing and curing methods, play a pivotal role in achieving optimal mechanical properties and precise fitting of the aligners [9,10].

The conversion rate in additive manufacturing significantly influences the final properties of printed objects, especially when it comes to versatile materials like polyurethane. Wienen et al. [11], as well as Moon et al. [12], stated that polyurethane’s extensive range of applications spans various industries, from creating intricate, biocompatible components in the medical field to serving as a durable, flexible option in automotive manufacturing. Its adaptability and ability to display properties ranging from those of soft elastomers to those of hard plastics make it a preferred choice in various sectors.

In additive manufacturing technologies like stereolithography (SLA) and digital light processing (DLP), which employ UV light to cure liquid resin layer by layer, achieving a high conversion rate is imperative. The conversion rate, indicating the degree of polymerization, directly correlates with the material’s performance characteristics [13]. Proper control over the polymerization process is crucial to tailoring polyurethane’s properties to specific application requirements [14]. 

When considering shape-memory polyurethane aligners, the conversion rate’s impact on the material’s mechanical properties becomes even more paramount. The material’s strength, elasticity, and durability depend on how effectively monomers or oligomers are converted into the polymer matrix. A higher conversion rate generally results in a denser, more cross-linked polymer network, enhancing the material’s strength and durability, which is crucial for aligners’ functionality. Incomplete conversion, however, can lead to defects and compromised mechanical integrity, affecting aligners’ performance and lifespan [15,16].

The polymerization conditions and post-processing protocols are intricately linked to achieving an optimal conversion rate. Oxygen’s presence during polymerization can inhibit the process, leading to lower conversion rates [17]. This is particularly concerning in air-based polymerization environments, where exposure to oxygen can prevent full material conversion, hindering aligners’ effectiveness [18]. In particular, the well-studied oxygen inhibition zone phenomenon acts as a barrier to the curing process under an open atmosphere [19]. Since 3D-printed aligners are constructed in a layer-upon-layer fashion, exposure to air during material setting may influence the degree of double bond conversion [20], potentially resulting in residual uncured free resin monomer and adversely affecting the mechanical properties of the aligner.

To complete the polymerization process effectively, 3D-printed objects must undergo a rinsing phase with a solvent to eliminate any residual unpolymerized resin, followed by placement in a chamber for light polymerization [20,21]. Various research endeavors [22,23,24,25,26] have delved into the post-curing methods that are applicable to additive manufacturing, specifically when it comes to temporary prostheses and materials for denture bases, underscoring the influential role of post-curing on the materials’ mechanical attributes. A prior study conducted by Li et al. [22] highlighted the benefits of stroboscopic post-curing within a nitrogen gas (N2) atmosphere, demonstrating its capacity to enhance the degree of conversion in materials utilized for 3D-printed temporary restorations. Nitrogen is widely utilized to create an oxygen-devoid environment in numerous industrial and chemical processes, including the curing and polymerization of materials like polyurethane, due to its abundance, cost-effectiveness, and inert nature. As the most abundant gas in Earth’s atmosphere, nitrogen is readily available and more economical than other inert gases, making it a practical choice for large-scale applications. Its chemical inertness is crucial for minimizing unwanted reactions during sensitive processes such as polymer curing, where oxygen can inhibit polymerization by reacting with essential free radicals. Research has shown that using nitrogen-rich, oxygen-free post-curing chambers impacts mechanical properties such as elasticity, hardness, and surface roughness [20,27,28]. These changes in properties can influence several aspects of an aligner’s performance, including the force it exerts, its relaxation behavior, its appearance, and its wear resistance [29].

While there is a growing body of work examining the mechanical properties of materials used in the additive manufacturing of occlusal splints [30,31], there remains a significant gap in the literature regarding how post-curing methods influence the mechanical properties of orthodontic aligner materials.

Post-processing steps, including rinsing protocols, significantly influence the conversion rate. The solvent choice, its interaction with polyurethane, and its effectiveness in removing uncured resin and impurities are crucial factors, as stated by Gomez et al. [32]. Ethanol, a less aggressive solvent compared to isopropanol, has shown promise in preserving the material’s integrity during the post-processing of PMMA materials [33]. When combined with an inert nitrogen polymerization environment, it contributes to higher conversion rates, potentially enhancing the aligner’s performance [34].

Given the specific demands of shape-memory polyurethane aligner materials, understanding the intricate relationship between post-processing protocols, polymerization conditions, and their cumulative impact on the conversion rate and mechanical properties of aligners becomes vital. This knowledge is crucial not only for optimizing the additive manufacturing process for polyurethane-based materials but also for ensuring that final printed aligners meet the required performance criteria, maintaining structural integrity and functionality throughout their use. The present study aims to shed light on these aspects, providing valuable insights that could guide the development and manufacturing of more effective, reliable polyurethane aligner materials.

## 2. Materials and Methods

### 2.1. Specimen Preparation

Rectangular specimens measuring 10 × 40 × 1 mm and 40 × 40 × 10 mm were made from Tera Harz TC-85 resin (Graphy, Seoul, Republic of Korea), a liquid resin based on polyurethane, to comply with international testing standards for different mechanical properties. This resin, designed for use in direct light processing technology, is printed in successive layers of 100 μm nominal size. After the additive manufacturing phase, the shape-memory polyurethane polymers derived from this resin undergo specific post-processing and post-curing treatments to develop the requisite mechanical characteristics and performance. The choice for two distinct sizes is aligned with the specifications set forth by ISO standards for various tests: the slimmer specimens (10 × 40 × 1 mm) conform to ISO 178:2019 [35], which is used for flexural testing to assess the material’s behavior under bending stress, while the larger specimens (40 × 40 × 10 mm) adhere to ISO 2039-1 [36], which dictates the requirements for hardness testing by the Ball Indentation Method. The printing process conducted on SprintRay Pro 95 (SprintRay, Los Angeles, CA, USA) was carefully monitored to ensure uniformity and adherence to the specified dimensions. Parameters such as layer height, print speed, and temperature were optimized to achieve the best possible results.

### 2.2. Post-Processing Protocols

**Figure 1:**
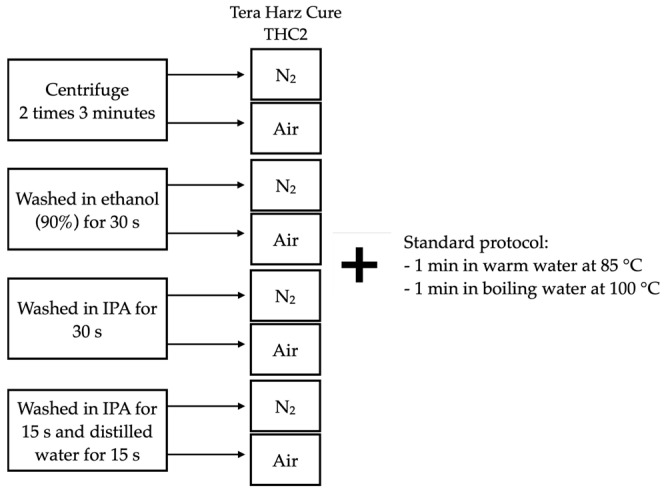
Post-processing protocols; IPA—isopropanol; air—ambient air; N_2_—nitrogen (oxygen-devoid environment).

Following the printing process, the specimens were subjected to one of eight post-processing protocols, tailored to enhance their material properties and ensure their readiness for subsequent testing (Figure 1).

Each protocol serves to improve the overall finish, mechanical integrity, and cleanliness of the printed objects, ensuring they meet the required standards for various applications, while also potentially reducing the presence of defects that could compromise their functionality and longevity [37,38,39,40,41,42,43,44].

### 2.3. Polymerization Process

Post-cleaning, the specimens were further polymerized in the Tera Harz Cure THC2 (Graphy, Seoul, Republic of Korea) to enhance their material properties. This step was conducted in two different environments (Figure 1):

1. Ambient air: The specimens were left to cure in a controlled environment with regular atmospheric conditions;
2. Oxygen-devoid environment: In this setting, the specimens were placed in a Tera Harz Cure THC2 (Graphy, Seoul, Republic of Korea) with a generator of nitrogen to inhibit oxygen, to facilitate a different polymerization process, potentially leading to variations in the material properties.

### 2.4. ATR-FTIR Analysis

FTIR spectra were recorded on Bruker Alpha ATR-FTIR spectrometer with diamond as a single-reflection crystal material. The spectra were collected in the wavelength range 4000–400 cm^−1^ with 10 scans at a resolution of 4 cm^−1^. Every sample was recorded at 12 different positions. For the instrument control as well as spectra manipulations (baseline correction, spectra normalization, automatic determination of band wavenumbers), OPUS 7.0 software was used. To improve the signal-to-noise ratios of the peaks selected for determining the degree of conversion, second derivative spectra were calculated using the Savitzky–Golay algorithm.

### 2.5. Mechanical Testing

#### 2.5.1. Flexural Modulus

To assess the flexural modulus of the materials, a three-point bending test was conducted using the precision Mark-10 testing machine (Mark-10 Corporation, Copiague, NY, USA), equipped with the IntelliMESUR^®^ software version 2.1.3. The machine was calibrated before testing to ensure accuracy and reliability in the results. Specimens (dimensions 40 × 10 × 1 mm) were placed on two supports with a span of 8 mm and a force was applied to the center of the specimen with a speed of 1 mm/s and end deflection of 5 mm. The load and displacement data were recorded and analyzed using the IntelliMESUR^®^ software to calculate the elastic modulus (ISO 178:2019) [35].

#### 2.5.2. Hardness Measurement

The materials’ hardnesses were quantitatively measured by the Ball Indentation Method on 40 × 40 × 1 mm specimens. In this procedure, a hardened steel ball with a 5 mm diameter was pressed into the surface of the specimen using a hardness tester machine (Zwick Roell, Ulm, Germany). The depth of the indentation was measured at 10 and 30 s, and the hardness value was calculated based on the applied load and the size of the indentation. This process was repeated 10 times for each specimen to ensure consistency and reliability in the results (ISO 2039-1) [35]. 

### 2.6. Statistical Analysis

The data analysis was performed using IBM SPSS Statistics software, version 29.0.1.0 (IBM, New York, NY, USA). QQ plots, the Shapiro–Wilk test, and calculations of skewness and kurtosis were used to check the normality of the data. Since the degree of conversion and flexural modulus were normally distributed, their descriptive statistics were expressed using mean and standard deviation and analyzed with a two-way ANOVA test, followed by Bonferroni post hoc correction. Hardness data, on the other hand, were analyzed using the Kruskal–Wallis test with Dunn’s post hoc test. The significance level was set at a *p*-value of 0.05.

## 3. Results

**Figure 2:**
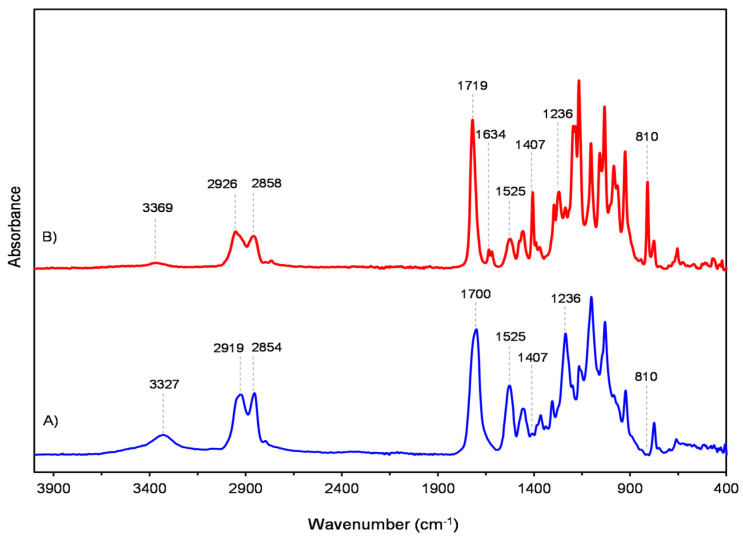
FTIR spectra of (**A**) liquid resin and (**B**) polymerized material.

FTIR spectra of liquid resin and polymerized material obtained by the standard protocol are shown in Figure 2. 

The FTIR spectrum of liquid resin revealed the presence of polyurethane and acrylate compounds. The spectrum is characterized by broad N–H stretching vibrations with the maximum being at 3369 cm^−1^, while the N–H bending deformation is visible as a band at 1525 cm^−1^. The sharp peak at 1719 cm^−1^ originates from the stretching vibration of nonhydrogen-bonded carbonyl groups of both compounds. The presence of acrylate is also indicated by a split peak with maxima at 1634 cm^−1^ and 1619 cm^−1^ as well as a peak observed at 810 cm^−1^ that corresponds to the stretching and twisting vibrations of aliphatic C=C groups, respectively. Vibrations in the range of ~1500–1350 cm^−1^ are attributed to symmetric and antisymmetric deformations of methyl and methylene groups. In this range, the dominant medium band at 1407 cm^−1^ is assigned to bending of the CH 2 = group. These groups are also characterized by both the antisymmetric and symmetric stretching of C–H bonds, which appear as weak bands in the region of ~3030–2815 cm^−1^. Below 1350 cm^−1^ up to 1000 cm^−1^, the spectrum is rich with vibrational modes of C–O–C ester bonds, among which a weak urethane C-O stretching band is present at 1236 cm^−1^ [45,46]. The spectrum of the aged polymeric material shows changes indicating that polymerization has occurred. Basic differences related to characteristic urethane –NH–CO–O– groups are due to the formation of hydrogen bonds. Namely, hydrogen bonding is responsible for the intensity increasing and a red shift of N–H stretching vibrations to 3327 cm^−1^, as well as for broadening of the C=O stretching band, which consists of two overlapped maxima associated with non-hydrogen and hydrogen bonded carbonyls. There is also a significantly increasing urethane C–O stretching band at 1236 cm^−1^. According to several studies, the quantitative aspect of monomer to polymer conversion was monitored by measuring the intensities of bands at 1634 cm^−1^ and 810 cm^−1^, originating from stretching and twisting vibrations of carbon-carbon acrylate double bonds [47,48,49]. Since the double vinyl group changes into a single C–C bond during the cross-linking reaction, it was expected that these bands would be highly diminished or completely absent in aged polymer [49]. The resolution of the band at 1634 cm^−1^ was improved by the calculation of second derivative spectra using the Savitzky–Golay algorithm. The degree of conversion (DC) is calculated from equation:

DC(%) = (A_P_ − A_LR_)/A_LR_ × 100

where A_LR_ and A_P_ are the absorbance intensities in liquid resins and after polymerization, respectively.

### 3.1. Degree of Conversion

**Figure 3:**
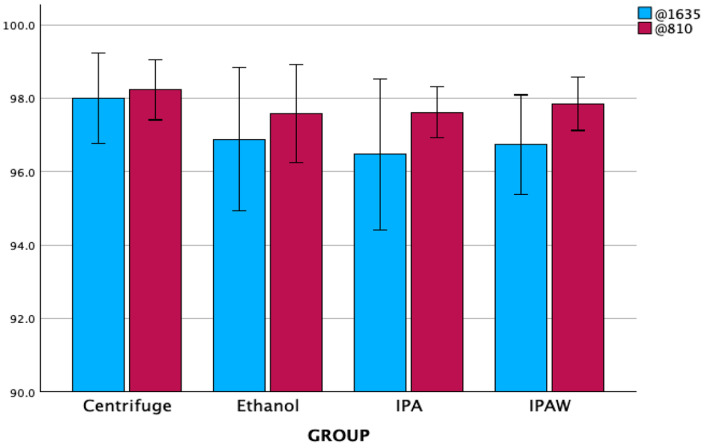
The degree of conversion (%) on two absorbance peaks 810 and 1635 cm^−1^; mean and SD.

**Figure 4:**
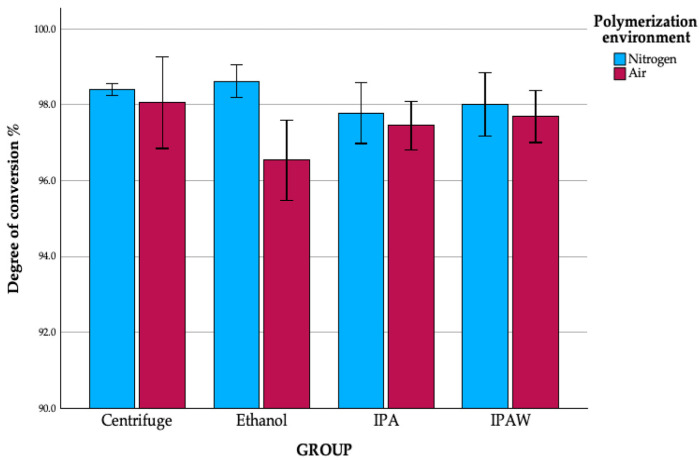
The degree of conversion (%) of different groups; mean and SD.

The standard protocol, comprising centrifugation followed by polymerization in a nitrogen environment, served as the baseline for our comparisons. The degree of conversion was measured on two absorbance peaks, at 810 and 1635 cm^−1^. Since there was a statistically significant difference in the degree of conversion measured, we used 810 because 1635 exhibited lower values with higher SD values (Figure 3). The highest conversion rate was noticed in the standard protocol group (98.4, SD 0.163), while the lowest rate was observed in the ethanol + air group (96.54, SD 1.057). In the investigation of the degree of conversion across the different groups studied, it was determined that there were no statistically significant differences in the degree of conversion among them (Table 1 and Figure 4). However, the polymerization environment had a quantifiable impact, accounting for 24.0% (partial eta squared = 0.240) of the variance in the degree of conversion (*p* = 0.011), which indicates that, while the degree of conversion was consistent across the groups, the environment in which polymerization was conducted played a significant role in influencing this aspect of the materials’ properties.

### 3.2. Flexural Modulus

**Figure 5:**
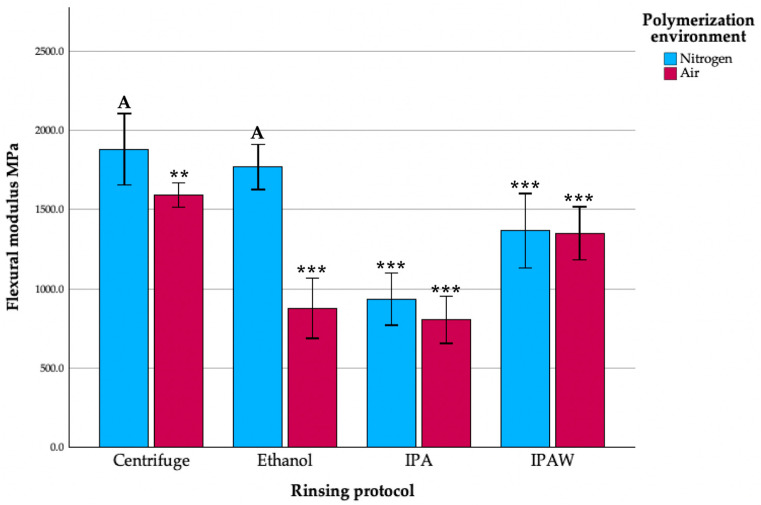
Flexural modulus (MPa) of different groups; letter A indicates statistically homogenous data with the standard protocol (Centrifuge + N2); ** *p* < 0.01, *** *p* < 0.001.

In conducting a comprehensive investigation to understand the impacts of post-processing protocols and polymerization environments on the flexural modulus of Tera Harz TC-85 resin, a two-way ANOVA was employed. The analysis aimed to discern the interaction between and main effects of rinsing groups and polymerization environments, using partial eta-squared values as a measure of the effect size. The interaction between the rinsing group and the polymerization environment yielded a substantial effect on the flexural modulus, with a partial eta squared value of 0.638, indicating that both factors collectively play a significant role in determining the material properties of the resin (Figure 5). The rinsing group alone accounted for a larger portion of the variance in the flexural modulus, with a partial eta squared value of 0.773, demonstrating its paramount importance in influencing the mechanical characteristics of the material. On the other hand, the polymerization environment held a partial eta squared value of 0.247, highlighting its substantial influence on the final material properties, albeit to a lesser extent than the rinsing group. When compared to the standard protocol of centrifugation followed by nitrogen polymerization, all of the other rinsing groups exhibited a statistically significantly lower flexural modulus, except for the ethanol + nitrogen group, which did not show a statistically significant deviation. The control group, centrifuge + nitrogen, exhibited the highest flexural modulus at 1881.22 MPa with a standard deviation of 224.48, serving as a testament to the efficacy of this protocol in optimizing material properties. On the other end of the spectrum, the group that underwent isopropanol rinsing followed by air polymerization recorded the lowest flexural modulus at 805.12 MPa, with a standard deviation of 149.6. These results underscore the significant impact of both the rinsing protocol and the polymerization environment on the flexural modulus of the studied material, necessitating optimal post-processing and polymerization conditions for superior material performance.

### 3.3. Hardness (Ball Identation Method)

**Figure 6:**
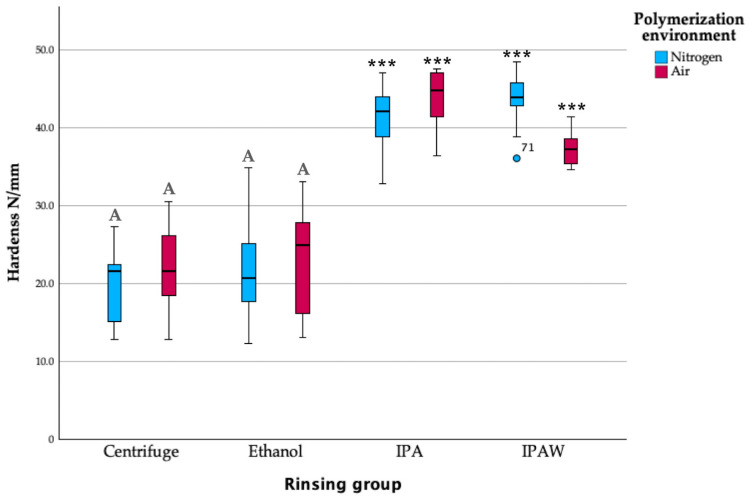
Hardness (N/mm) of different groups at 10 s; letter A indicates statistically homogenous data with the standard protocol (Centrifuge + N2); *** *p* < 0.001.

**Figure 7:**
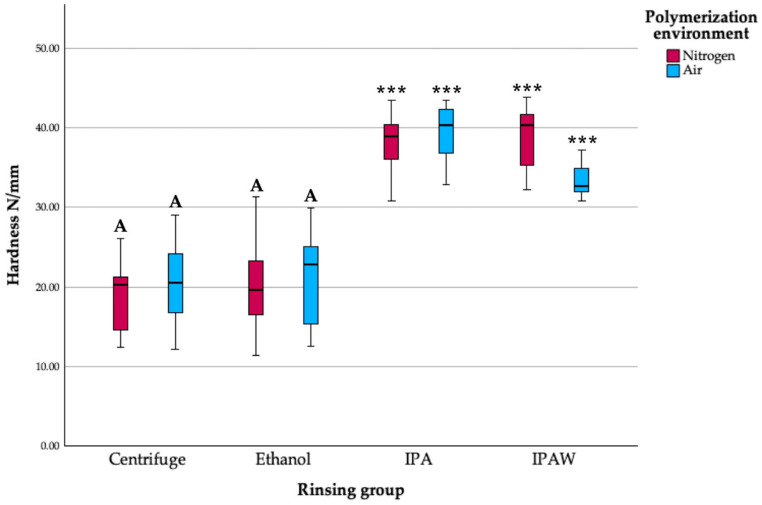
Hardness (N/mm) of different groups at 30 s; letter A indicates statistically homogenous data with the standard protocol (Centrifuge + N2); *** *p* < 0.001.

In evaluating the hardness of Tera Harz TC-85 resin samples, the Ball Indentation Method was employed for 10 s and 30 s, providing a precise and consistent measure of the material’s resistance to deformation (Figure 6 and Figure 7). Through analysis, it was observed that there was no statistically significant difference in hardness value between the control group (centrifuge + nitrogen) and the centrifuge + air group, indicating that shifting from an inert nitrogen environment to an air environment post-centrifugation did not notably alter the material’s hardness. Similarly, the ethanol + air and ethanol + nitrogen groups demonstrated consistency in their hardness values, showing no statistically significant differences between them and implying that the ethanol rinsing protocol resulted in a uniform hardness characteristic across different polymerization environments. However, a stark contrast emerged when these groups were compared to the IPA and IPAW groups, as the latter consistently displayed higher hardness values, a difference that was found to be statistically significant. This highlights the considerable impact of the rinsing protocol on the material’s hardness, with IPA treatment leading to enhanced resistance to deformation compared to the other groups.

## 4. Discussion

In the present study, the comparison of different post-processing protocols and polymerization environments has yielded a comprehensive understanding of their impacts on the conversion rate of Tera Harz TC-85 resin, a polyurethane-based material.

Our analysis of the Tera Harz TC-85 resin, a product known for its advanced applications in additive manufacturing, reveals intriguing insights into its chemical composition. Our FTIR analysis suggests the presence of both polyurethane and acrylate compounds within the resin formulation. Polyurethane is identifiable in the spectrum by its characteristic absorption bands, typically attributed to the urethane linkages, which include N–H stretching and C=O (carbonyl) stretching vibrations [50]. These bands are indicative of the flexible and durable nature of polyurethane, which contributes to the resin’s mechanical properties like elasticity and toughness. On the other hand, the presence of acrylate compounds is revealed by the distinct peaks associated with C=C stretching vibrations in the acrylate groups [46]. These acrylate components are crucial for the resin’s UV-curing capabilities, a property harnessed in additive manufacturing for rapid solidification under UV light. The coexistence of polyurethane and acrylate in the Tera Harz TC-85 resin suggests a hybrid formulation that aims to combine the beneficial properties of both materials. This hybrid composition could potentially offer the structural integrity and flexibility of polyurethanes, along with the quick-curing properties of acrylates, making the resin suitable for a wide range of additive manufacturing applications [51,52]. Furthermore, the integration of acrylate with polyurethane in the resin matrix could also enhance the final product’s surface finish and detail resolution, an aspect critical in high-precision additive manufacturing applications [53]. 

The evident decrease in the conversion rate across all rinsing groups when polymerized in an air environment, compared to the standard protocol of centrifugation followed by polymerization in a nitrogen environment, underscores the detrimental effects of oxygen on the polymerization process. The safety profile of nitrogen, being non-toxic and non-flammable, also contributes to its widespread use [54]. While vacuum environments can also remove oxygen, their application can be more complex and costly, especially for continuous processes, and not all materials or processes respond well to the low-pressure conditions of a vacuum [34]. Other inert gases like argon, while offering a higher degree of inertness, are often more expensive and may not provide significant advantages over nitrogen for many applications [55]. Therefore, nitrogen remains a preferred choice for creating an oxygen-free environment in various industrial and laboratory settings, balancing cost, effectiveness, and safety.

The recent literature has increasingly focused on the phenomenon of oxygen inhibition in the polymerization of polymer resins, highlighting it as a critical challenge in the field of polymer science and additive manufacturing [56,57,58,59,60]. A study by Jung-Hwa et al. has detailed how oxygen, particularly in ambient air, can interfere with the curing process of photopolymer resins, leading to incomplete polymerization and compromised material properties [34]. This is particularly pertinent in UV-cured resins, where oxygen can quench the free radicals necessary for polymerization [14,61]. Furthermore, various strategies have been investigated to mitigate oxygen inhibition, including the use of inert atmospheres during curing and the development of oxygen-scavenging additives [62,63]. These approaches aim to enhance the degree of conversion and mechanical properties of the resin, a goal echoed in newer studies that examined the effects of reduced oxygen environments on the tensile strength and surface roughness of 3d-printed objects [64,65]. The results are in alignment with previous studies, which have highlighted the negative impact of oxygen on the polymerization of polyurethane-based resins, emphasizing the need for an oxygen-devoid environment to achieve optimal results [17,61,62,66,67].

The standard protocol of centrifuge + nitrogen, serving as a control, showcases the importance of eliminating oxygen from the polymerization environment. This aligns with previous findings, which demonstrated that polyurethane-based materials exhibit enhanced mechanical properties and higher conversion rates when polymerized in inert environments [32,37,68]. Building upon the insights gathered from the different post-processing protocols and polymerization environments, it is intriguing to delve into why ethanol rinsing followed by nitrogen polymerization provided results akin to the standard protocol.

Ethanol, being a less reactive solvent compared to others like isopropanol, may play a role in preserving the integrity of the polyurethane material during the post-processing phase. One of its notable properties is its relatively fast evaporation rate, which enables easy removal from the printed object post-rinsing [68]. This characteristic is crucial, as it ensures that no residue remains on the object which could otherwise interfere with subsequent polymerization steps [68]. Moreover, ethanol is considered a safer option compared to many other organic solvents due to its lower toxicity levels. Its safety profile, combined with its wide availability and cost-effectiveness, makes ethanol a practical and preferred choice in both industrial and laboratory settings for the post-processing of 3d-printed materials. Additionally, ethanol’s relatively low reactivity ensures that the material’s conversion process is not adversely affected during the rinsing phase. Ethanol might interact with the resin matrix in a manner that promotes better cross-linking when devoid of oxygen. When ethanol-rinsed specimens are cured in a nitrogen atmosphere, it may provide an environment conducive to optimal curing, promoting a more organized and dense polymer network [69]. Subsequent polymerization in a nitrogen environment ensures that the material is not exposed to oxygen, which, as previously discussed, can act as an inhibitor to the polymerization process [17,61,62,66,67]. This inert atmosphere promotes a more complete conversion, allowing the material to reach its optimal mechanical properties. This study showed that the combination of ethanol rinsing and nitrogen polymerization created a conducive environment for the polyurethane material to achieve a high conversion rate, similar to the standard protocol. In light of the conversion rates discussed earlier, these findings further reinforce the importance of carefully selecting post-processing protocols to optimize material properties. The fact that the ethanol + nitrogen group did not significantly differ from the standard protocol in flexural modulus, hardness, or degree of conversion demonstrates that there are alternative post-processing routes that can achieve similar outcomes, broadening the options available for material processing and potentially paving the way for more efficient and tailored manufacturing workflows. 

The significantly higher hardness values in groups rinsed with IPA and IPAW can be associated with the solvent’s characteristics. IPA is known to be a more aggressive solvent compared to ethanol [33], potentially leading to a more extensive removal of uncured resin and potentially aiding in better cross-linking within the material during polymerization. This could result in a denser material, contributing to the increased hardness which was observed. The presence of water in the IPAW group might further facilitate this process, as water is known to react with isocyanate groups in polyurethane, potentially leading to additional cross-linking and resulting in a harder material [70]. The consistent hardness values observed among the control group (centrifuge + nitrogen) and both ethanol + air and ethanol + nitrogen groups offer key insights into the influence of ethanol rinsing on Tera Harz TC-85 resin. These results suggest that the ethanol rinsing protocol yields a uniform hardness characteristic, irrespective of whether the polymerization environment is air or nitrogen. This uniformity indicates that ethanol rinsing effectively stabilizes the hardness properties of the resin, making it less susceptible to variations due to different post-polymerization atmospheres. These findings can significantly inform material processing strategies, especially in applications where uniform hardness is desired. For instance, manufacturers can opt for ethanol rinsing as a reliable step to ensure a consistent material quality across various production environments. Additionally, understanding that ethanol rinsing maintains hardness properties irrespective of air or nitrogen atmospheres provides flexibility in choosing polymerization environments without compromising the mechanical properties of the resin. 

The divergence observed in the results, with the control group and ethanol + nitrogen group showing the highest values in their conversion rate and flexural modulus while possessing lower hardness values in comparison to the IPA and IPAW groups, is a complex interplay of material characteristics influenced by post-processing protocols and polymerization environments. The enhanced conversion rate and flexural modulus in the control and ethanol + nitrogen groups can be attributed to the nitrogen environment minimizing the inhibitory impacts of oxygen, ensuring a more complete and uniform conversion, which is crucial for achieving optimal mechanical properties [22]. Ethanol, being a mild solvent, preserves the material’s integrity, which is vital in facilitating these optimal conditions for material conversion, directly influencing the flexural modulus and indicating a material that is stiffer and more resistant to deformation under stress [68]. This, in the context of 3D-printed shape-memory polyurethane materials, translates to aligners that are robust in delivering a consistent and predictable force, a prerequisite for effective teeth movement [4,71,72]. However, this advantage seems to be at the expense of hardness. The gentler nature of ethanol, while preserving the material integrity, results in a lower material density or lower cross-linking in comparison to the IPA-treated groups, leading to lower hardness values [40,73]. On the other hand, IPA, known for its aggressive solvent nature, enhances material densification and cross-linking, as is evident from the higher hardness values observed in the IPA and IPAW groups [40]. While these groups might boast enhanced wear resistance due to their higher hardnesses, they may not offer the same level of performance predictability due to potential compromises in their conversion rate and flexural modulus. In practical terms, for aligners, this could mean a material that, while potentially more resistant to surface wear, might not maintain its intended shape and force delivery over time, an essential aspect for controlled teeth movement. In a hybrid resin comprising both polyurethane and acrylate components, the interaction between these two factors—the rinsing process and the polymerization environment—can be complex. The rinsing protocol may predominantly alter the characteristics of the polyurethane, while the polymerization environment may more significantly influence the properties of the acrylate. However, due to the interconnected nature of the hybrid network, changes in one component could indirectly influence the other, affecting the overall material properties of the resin. This intricate balance between material properties underscores the complexity of material selection and optimization for specific dental applications, highlighting the necessity of a holistic approach to material characteristics to achieve a harmonious balance between performance, comfort, and wear resistance [74,75,76,77]. 

## 5. Conclusions

Based on the comprehensive results presented, the key conclusions are:

1. The FTIR spectra indicated the presence of polyurethane and acrylate compounds in both liquid and polymerized Tera Harz TC-85 resin, with distinct changes in N–H, C=O, and C=C stretching vibrations due to polymerization;
2. The highest degree of conversion was observed in the standard protocol (98.4%), with no significant differences across groups. However, the polymerization environment significantly influenced the degree of conversion (η_p_^2^ = 0.240, *p* = 0.011);
3. The rinsing protocols and polymerization environments substantially affected the flexural modulus (η_p_^2^ = 0.638, *p* < 0.001), with the standard protocol showing the highest modulus and isopropanol rinsing with air polymerization yielding the lowest;
4. Hardness measurements showed no significant difference among the control and centrifuge + air groups as well as the ethanol rinsing groups, while IPA treatments led to a significantly higher hardness compared to other groups.

The detrimental impact of oxygen on the polymerization process was clearly demonstrated, underscoring the necessity of an oxygen-devoid environment for obtaining optimal material properties. Interestingly, the ethanol + nitrogen group presented itself as a viable alternative to the standard centrifuge + nitrogen protocol, achieving comparable results in terms of the conversion rate and flexural modulus while maintaining a lower hardness level.

## Figures and Tables

**Table 1:** The degree of conversion (%) of different groups; CL—confidence limit.

		Mean	95.0% Lower CL for Mean	95.0% Upper CL for Mean	Standard Deviation
Centrifuge	Nitrogen	98.40	98.14	98.66	0.16
	Air	98.05	96.13	99.96	1.21
Ethanol	Nitrogen	98.62	97.93	99.31	0.43
	Air	96.54	94.86	98.22	1.06
IPA	Nitrogen	97.78	96.50	99.07	0.81
	Air	97.45	96.43	98.46	0.64
IPAW	Nitrogen	98.00	96.67	99.33	0.84
	Air	97.69	96.59	98.78	0.69

## Data Availability

The data presented in this study are available on request from the corresponding author.
